# Literary Notes

**Published:** 1902-03

**Authors:** 


### LITERARY NOTES.

The Vermont *Medical Monthly* began the new year with a complete change in style and dress. It is now a double columned quarto and makes a handsome appearance in its new clothes.

---

The *Woman's Medical Journal*, published at Toledo, O., has changed its form and taken on a new dress. It is now a double-columned quarto and presents a prosperous appearance. Dr. Elizabeth Dunn has been added to its editorial staff.

---

The Nathan Lewis Hatfield prize for original research in medicine by the College of Physicians of Philadelphia announces through its committee that the sum of $500 will be awarded to the author of the best essay in competition for the above prize. Subject, The relation between chronic suppurative processes and forms of anemia. Essays must be submitted on or before March 1, 1903. The details can be obtained upon application to Dr. J. C. Wilson, chairman, 219 South 13th Street, Philadelphia, Pa.

---

THE *Cleveland Journal of Medicine* and the *Cleveland Medical Gazette*, have united their interests, good names, and subscription lists in one magazine to be called the *Cleveland Medical Journal*. The new journal is owned and published by the Cleveland Medical Journal Company, whose officers are as follows: *President*, Dr. Marcus Rosenwasser; *Vice-President*, ————; *Secretary*, Dr. William E. Bruner; *Treasurer*, Dr. Joseph F. Hobson; *Editor*, Dr. P. Maxwell Foshay; *Associate Editor*, Dr. Edward S. Lander. The stockholders mumber about 45 of Cleveland's representative physicians, who announce that they do not expect dividends. We believe the amalgamation a wise act, and bespeak for our new contemporary a prosperous and happy career.

